# Impact of first-line protease inhibitors on predicted resistance to tipranavir in HIV-1-infected patients with virological failure

**DOI:** 10.1186/1471-2334-9-154

**Published:** 2009-09-14

**Authors:** Szu-Min Hsieh, Sui-Yuan Chang, Chien-Ching Hung, Wang-Huei Sheng, Mao-Yuan Chen, Shan-Chwen Chang

**Affiliations:** 1 Department of Internal Medicine, National Taiwan University Hospital and National Taiwan University College of Medicine, Taipei, Taiwan, Republic of China; 2 Department of Laboratory Medicine, National Taiwan University Hospital and National Taiwan University College of Medicine, Taipei, Taiwan, Republic of China

## Abstract

**Background:**

Tipranavir (TPV) is a recently approved nonpeptidic protease inhibitor (PI) of HIV-1 and has been indicated for those infected with PIs-resistant HIV-1. However, in clinical practice, whether the HIV-1 from the patients with virological failure to the regimens containing first-line PIs remains susceptible to TPV/r may be questionable.

**Methods:**

To assess the resistance levels to TPV of HIV-1 from patients with treatment failure to first-line PIs, patients who experienced virological failure were tested for genotypic resistance of HIV-1 since August 2006 in National Taiwan University Hospital. Patients were enrolled for this analysis if their failed regimens contained > 12 weeks of atazanavir or lopinavir/ritonavir (defined as ATV group and LPV/r group, respectively), but were excluded if they experienced both or other PIs. The levels of genotypic resistance to TPV/r were determined by TPV mutation score.

**Results:**

Till May 2008, 21 subjects in ATV group and 20 subjects in LPV/r group were enrolled. The TPV mutation scores in subjects in LPV/r group were significantly higher than these in ATV group (median, 3 vs 1, P = 0.007). 95.2% subjects in ATV group and only 45% subjects in LPV/r group had an estimated maximal virological response to TPV/r (P < 0.001). The resistance levels to TPV/r correlated with the duration of exposure to first-line PIs, whether in ATV or LPV/r group.

**Conclusion:**

Cross-resistance from first-line PIs may impede the effectiveness of TPV/r-containing salvage therapy. TPV/r should be used cautiously for patients with virological failure to LPV/r especially long duration of exposure.

## Background

Tipranavir (TPV) is a recently approved nonpeptidic protease inhibitor (PI) of HIV-1 and ritonavir (RTV)-boosted tipranavir (TPV/r) has been indicated for treatment-experienced patients or those infected with PIs-resistant HIV-1 [1-3] thus TPV/r is only approved in highly treated patients with a documented resistance to multiple PIs in Taiwan.

However, TPV shares some resistance-associated mutations (such as M36I, M46L, I54V, I84V, etc) with other PIs [4]. Thus, in clinical practice, whether the HIV-1 derived from the patients with virological failure to the regimens containing first-line PIs remains susceptible to TPV/r may be questionable. RTV-boosted lopinavir (LPV/r) and atazanavir (ATV) are recommended as the preferred first-line PIs for antiretrovirals-naïve patients [5], therefore we assessed and compared the levels of TPV resistance of HIV-1 from patients with virological failure to the ATV or LPV/r-containing antiretroviral regimens. Because resistance testing is not necessarily feasible in areas where second-line antiretrovirals are available, these data may help to decide the adequate role and timing of initiating TPV/r-containing salvage therapy.

## Methods

### Study population

Since August 2006, HIV-1-infected patients who experienced virological failure were tested for genotypic resistance of HIV-1 in National Taiwan University Hospital, the major referral center for HIV/AIDS and reference laboratory for HIV-1 resistance testing in Taiwan. Virological failure was defined if a confirmed HIV RNA level > 400 copies/mL after 24 weeks of antiretroviral treatment, or > 50 copies/mL after 48 weeks, or repeated detectable HIV RNA level after prior suppression of viremia. Resistance testing was performed while the patients were taking or immediately (< 4 weeks) after discontinuation of the failed regimen. Patients were enrolled for this analysis if their failed regimens contained > 12 weeks of LPV/r or ATV (defined as LPV/r group and ATV group, respectively), and were excluded from the analysis if they experienced both of LPV and ATV, or using any antiretrovirals more than 12 weeks prior to the first-line PIs, or if they had a plasma HIV RNA < 1000 copies/mL. Low-dose RTV was not counted as a separate drug. This study has been approved by the Institutional Review Board of the hospital and informed consents have been obtained from all of the subjects before analysis. Initiating LPV/r or ATV depends on doctors' choice.

### Genotypic resistance assay

This assay has been described previously [6]. Briefly, total RNA was extracted from plasma using the QIAamp Viral RNA Mini Kit (QIAGEN, CA, USA) according to the manufacturer's protocol. The PCR reaction was carried out in a final volume of 50 μL containing 20 mM Tris-HCl (pH 8.4), 50 mM KCl, 1.5 mM MgCl_2_, 0.2 mM each deoxynucleoside triphosphate, 0.2 μM of each specific primer, 2.5 U of platinum *Taq *DNA polymerase (Invitrogen Life Technologies, USA). Population-based nucleotide sequence analysis of the PCR fragments was conducted using an automatic sequencer (3100 Avent Genetic Analyzer, ABI, CA, USA).

### Tipranavir mutation score

We assessed the genotypic susceptibility of TPV/r by using a unweighted tipranavir mutation score as described by Baxter et al. in 2006 [7]. The score is determined by the number of indicated mutations, consisting of L10V, I13V, K20M/R/V, L33F, E35G, M36I, K43T, M46L, I47V, I54A/M/V, Q58E, H69K, T74P, V82L/T, N83D, I84V. An increasing point was associated with a higher level of resistance.

### Statistical analysis

Statistical significance was determined using a non-parametric test (Mann-Whitney *U *test) to compare the duration of exposure to PI, and using Fisher exact test or Chi-square test for categorical variables. Linear correlation was evaluated by Pearson's correlation coefficient. *P *< 0.05 was considered statistically significant.

## Results

### Characteristics of subjects

From August 2006 to May 2008, a total of 41 subjects met the criteria for this analysis: 21 subjects with virological failure to ATV-containing regimens (ATV group; five of them also received low-dose RTV for boosting ATV) and 20 subjects with virological failure to LPV/r-containing regimens (LPV/r group). The CD4+ cell counts, plasma HIV RNA, and the total duration of PI exposure at the time of genotypic testing are similar in ATV group vs. LPV/r group (Table 1). The percentages of subjects that had at least one mutation listed in the TPV mutation score were not significantly different in both groups (11/21 vs. 15/20, P = 0.197). Among these mutations, M36I was the most common mutation in both group (6/11 and 9/15, respectively).

**Table 1:** Selected characteristics of enrolled subjects in this study

	ATV/r (n = 21)	LPV/r (n = 20)	P value
Sex (M/F)	21/0	20/0	
Age: median, range (years)	33.5, 21-45	35, 22-57	0.53
CD4 cell count at baseline: median, range (per μgL)	132, 25-367	121, 18-340	0.37
Plasma HIV RNA at baseline: median, range (copies/mL)	35200, 7500-232000	28900, 3500-356000	0.42
CD4 cell count at virological failure: median, range (per μgL)	195, 37-468	202, 65-520	0.56
Plasma HIV RNA at virological failure: median, range (copies/mL)	26500, 1500-87300	22500, 2200-67200	0.37
History of AIDS: no. (%)	14 (67)	15 (75)	0.808
Experienced to NNRTI: no. (%)	12 (57)	13 (65)	0.845
Duration of PI exposure: median, range (months)	13.2, 4.8-18	11.3, 3.5-17	0.32

### Levels of genotypic resistance to TPV/r

**Figure 1:**
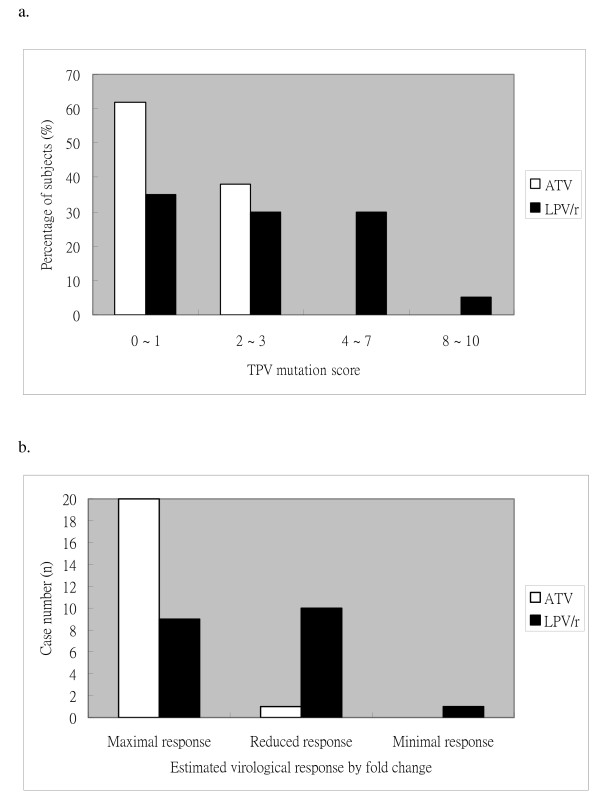
**Susceptibility to tipranavir**. a. Distribution of tipranavir mutation score: The distribution of tipranavir mutation score in subjects with virological failure to antiretroviral regimens containing ritonavir-boosted lopinavir (LPV/r, n = 20) or atazanavir (ATV, n = 21). P = 0.037 (by Chi-square test). b. Estimated virological responses to tipranavir: Assessment of the estimated virological responses to TPV/r in patients experiencing virological failure to regimens containing LPV/r or ATV. The maximal response is estimated by TPV score <2, reduced response by TPV score of 3-7, and minimal response by TPV score ≥ 8.

The TPV resistance levels in subjects in LPV/r group are significantly higher than these in ATV group (TPV mutation score, median, 3 vs 1, P = 0.007). Based on the study of Baxter et al, TPV mutation scores of ≤ 1 were associated with < 1 median fold change (FC) of IC50 from the wild-type susceptibility, the scores of 4~ 7 were associated with a median FC of 2 ~ 3.9, and the scores of > 8 were associated with a dramatic increase in median FC (> 14.7) [7]. We then compared the genotypic resistance of HIV-1 to TPV/r between LPV/r group and ATV group by categorizing the score points into 0~ 1, 2~ 3, 4~ 7, and ≥ 8 (Figure 1a). No one in ATV group had a score ≥ 4; however, among the 15 subjects in LPV/r group that had ≥ 1 mutation listed in the score, near half (7/15) had a score ≥ 4 (0/11 vs. 7/15, P = 0.01). These analyses showed significantly higher levels of genotypic resistance to TPV in subjects with virological failure with LPV/r-containing regimens. The TPV mutation score was not significantly different between the subjects with RTV-boosted ATV and the subjects with ATV without RTV.

### Estimated phenotypic resistance by fold change

Based on the Virco's algorithms, the lower and higher clinical cut-offs (CCO1 and CCO2, respectively) of FC from the wild-type susceptibility to TPV/r are 1.2 and 5.4, respectively [8-10]. FC ≤ CCO1 indicates an estimated maximal virological response, CCO1 < FC ≤ CCO2 indicates an estimated reduced response, and FC > CCO2 indicates minimal response. The CCO of 1.2 and 5.4 are correlated with TPV score of 2 and 8, thus the maximal response could be estimated by TPV score ≤ 2, reduced response by TPV score of 3-7, and minimal response by TPV score ≥ 8 [7] (Figure 1b). The results showed 20 subjects in ATV group (95.2%) and 9 subjects in LPV/r group (45%) had a TPV mutation score ≤ 2 (20/21 vs. 9/20, P < 0.001), that indicated most subjects after virological failure with ATV/r-containing regimen and only less than half subjects after virological failure with LPV/r-containing regimens may had an estimated maximal virological response to TPV/r-containing regimens

### Correlation of the resistance levels to TPV/r with the exposure duration to first-line PIs

**Figure 2:**
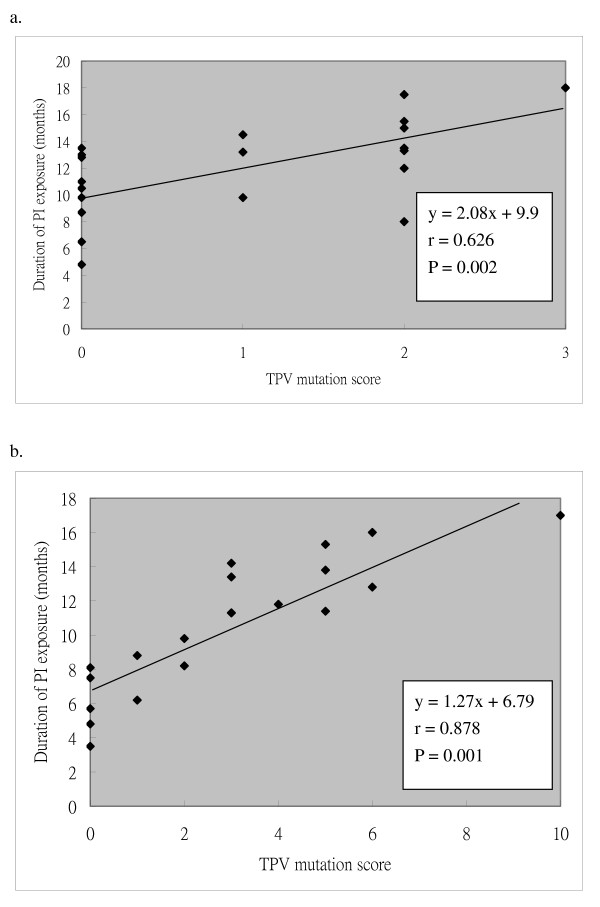
**Correlation between the exposure duration and the tipranavir mutation score**. Correlations between the duration of exposure to protease inhibitors in subjects with virological failure and the tipranavir (TPV) mutation score of HIV-1 derived from these subjects, were assessed. Linear correlation was evaluated by Pearson's correlation coefficient. a. PI = Atazanavir (ATV) ± ritonavir (RTV). b. PI = Lopinavir ± ritonavir (LPV/r).

To know whether longer durations of exposure to PIs are associated with higher levels of resistance to TPV/r, we assessed the relationship between the TPV mutation scores and the duration of exposure to first-line PIs in these subjects with virological failure to regimens containing first-line PIs. The data showed that the TPV mutation scores had a linear correlation with the duration of exposure to first-line PIs, especially in subjects in LPV/r group (Figure 2).

## Discussion

This study showed the HIV-1 derived from the subjects after virological failure with LPV/r-containing regimens had a significantly higher genotypic resistance to TPV/r, than that from the subjects after virological failure with ATV-containing regimens, under the similar duration of PI exposure. Furthermore, in these subjects with virological failure to regimens containing first-line PIs, the TPV resistance levels had a linear correlation with the duration of exposure to ATV or LPV/r, indicating accumulation of mutations through time. The results may indicate that the subjects with virological failure with LPV/r-containing regimens may less likely respond to TPV/r-containing regimens, thus TPV/r may not be an ideal empirical choice for patients with virological failure with LPV/r-containing regimens, especially in a long duration of exposure. The mechanisms remain to be investigated. The possible explanation is that LPV/r has a high genetic barrier for HIV-1 resistance; once HIV-1 develops resistance to LPV/r *in vivo*, extensive levels of resistance-associated mutations in HIV-1 protease gene could be identified [11].

Only three subjects had a TPV mutation score of ≥ 6 in LPV/r group. This could mean that most patients on a failing LPV/r-containing regimens may just have a reduced virological response but not necessarily virological failure when shifted to TPV-containing salvage regimens. The clinical success may still possibly be achieved by maximizing the number of active drugs in the background regimens and improving the adherence of patients.

The study has several limitations. The reason to explain why such high levels of resistance to TPV could be identified after virological failure to PI-containing regimens in a short duration of PI exposure in these subjects may include inadequate adherence or high baseline resistance. However, the impact of medication adherence on the resistance emergence, that may bias the interpretation for the results, could not be quantitatively assessed in this study. Though the resistance rate to PIs in treatment-naïve patients has been documented to have a significant increase in recent years in Taiwan [6], our data lack for the baseline resistance information of these subjects to exactly assess how many major mutations developed during the treatment with PI-containing regimens. Among the enrolled subjects in this study, the data of subtype distribution is not complete. Thus, we can not define the impact of subtypes on the treatment responses and emergence of the resistance-associated mutations. However, it appears that HIV-1 subtypes do not effect major differences in the response to antiretroviral therapy and in the mutations leading to resistance [12]. The study is also limited by small case number thus the findings should be validated by a large-scale randomized study.

## Conclusion

The subjects with virological failure to LPV/r-containing regimens may less likely respond to TPV/r-containing salvage regimens than these with virological failure to ATV/r-containing regimens, especially in a long duration of exposure. Even the feasibility of resistance testing is limited, the empirical use of TPV/r should be very cautiously in subjects with virological failure to LPV/r-containing regimens unless resistance assay showed no or few mutations associated with TPV resistance.

## Competing interests

The authors declare that they have no competing interests.

## Authors' contributions

SMH and SYC participated in its design and coordination and drafted the manuscript. CCH and WHS made substantial contributions to conception and design and acquisition of data and analysis and interpretation of data. MYC and SCC involved in drafting the manuscript and revised it critically for important intellectual content. All authors read and approved the final manuscript.

## Pre-publication history

The pre-publication history for this paper can be accessed here:

http://www.biomedcentral.com/1471-2334/9/154/prepub

## References

[B1] Temesgen Z Feinberg J Tipranavir: A New Option for the Treatment of Drug-Resistant HIV Infection Clin Infect Dis 2007 45 761 769 10.1086/520847 17712762

[B2] Markowitz M Slater LN Schwartz R Long-term efficacy and safety of tipranavir boosted with ritonavir in HIV-1-infected patients failing multiple protease inhibitor regimens J Acquir Immune Defic Syndr 2007 45 401 410 10.1097/QAI.0b013e318074eff5 17554217

[B3] Hicks CB Cahn P Cooper DA Durable efficacy of tipranavir-ritonavir in combination with an optimized background regimen of antiretroviral drugs for treatment-experienced HIV-1-infected patients at 48 weeks in the Randomized Evaluation of Strategic Intervention in multidrug reSistant patients with Tipranavir (RESIST) studies: an analysis of combined data from two randomized open-label trials Lancet 2006 368 466 475 10.1016/S0140-6736(06)69154-X 16890833

[B4] Johnson VA Brun-Vezinet F Clotet B Update of the drug resistance mutations in HIV-1: spring 2008 Top HIV Med 2008 16 62 68 18441382 10.1007/s11750-007-0034-z

[B5] Department of Health and Human Services (DHHS) panel Guidelines for the use of antiretroviral agents in HIV-1-infected adults and adolescents 2008 http://AIDSinfo.nih.gov 11364535

[B6] Chang SY Chen MY Lee CN Trend of antiretroviral drug resistance in treatment-naïve patients with HIV-1 infection in Taiwan J Antimicrob Chemother 2008 61 689 693 10.1093/jac/dkn002 18227088

[B7] Baxter JD Schapiro JM Boucher CA Genotypic changes in human immunodeficiency virus type 1 protease associated with reduced susceptibility and virologic response to the protease inhibitor tipranavir J Virol 2006 80 10794 10801 10.1128/JVI.00712-06 16928764 PMC1641746

[B8] Borght K Van der Winters B Van Craenenbroeck E Correlation of resistance algorithms for tipranavir susceptibility with response to tipranavir containing regimens in the RESIST trials Program and abstracts of the 5th European HIV Drug Resistance Workshop 2007 http://www.natap.org

[B9] Bacheler L Vermeiren H Winters B Clinically relevant phenotypic resistance and cross-resistance to tipranavir among recent routine clinical isolates Program and abstracts of the 4th European HIV Drug Resistance Workshop 2006 http://www.hivpresentation.com/

[B10] Villacian J van Craenenbroeck E Winters B Bacheler L Phenotypic cut-off values for the clinical interpretation of predicted viral fold change for antiretroviral agents: the vircoTYPE HIV-1 approach Presented at the 4th AS Conference on HIV Pathogenesis, Treatment and Prevention, Sydney, Australia, 22-25 July, 2007

[B11] Kempf D Brun S Rode R Identification of clinically relevant phenotypic and genotypic breakpoints for ABT-378/r in multiple PI-experienced, NNRTI-naive patients Antiviral Ther 2000 5 Suppl 3 70 75

[B12] Taylor BS Sobieszczyk ME McCutchan FE Hammer SM The Challenge of HIV-1 Subtype Diversity N Engl J Med 2008 358 1590 1602 10.1056/NEJMra0706737 18403767 PMC2614444

